# Petunia, Your Next Supermodel?

**DOI:** 10.3389/fpls.2016.00072

**Published:** 2016-02-02

**Authors:** Michiel Vandenbussche, Pierre Chambrier, Suzanne Rodrigues Bento, Patrice Morel

**Affiliations:** Laboratoire de Reproduction et Développement des Plantes, UMR5667 CNRS, INRA, ENS Lyon, Université de LyonLyon, France

**Keywords:** petunia, model system, genome sequence, transposon mutagenesis, functional genomics, evolution, plants, evo–devo

## Abstract

Plant biology in general, and plant evo–devo in particular would strongly benefit from a broader range of available model systems. In recent years, technological advances have facilitated the analysis and comparison of individual gene functions in multiple species, representing now a fairly wide taxonomic range of the plant kingdom. Because genes are embedded in gene networks, studying evolution of gene function ultimately should be put in the context of studying the evolution of entire gene networks, since changes in the function of a single gene will normally go together with further changes in its network environment. For this reason, plant comparative biology/evo–devo will require the availability of a defined set of ‘super’ models occupying key taxonomic positions, in which performing gene functional analysis and testing genetic interactions ideally is as straightforward as, e.g., in *Arabidopsis*. Here we review why petunia has the potential to become one of these future supermodels, as a representative of the Asterid clade. We will first detail its intrinsic qualities as a model system. Next, we highlight how the revolution in sequencing technologies will now finally allows exploitation of the petunia system to its full potential, despite that petunia has already a long history as a model in plant molecular biology and genetics. We conclude with a series of arguments in favor of a more diversified multi-model approach in plant biology, and we point out where the petunia model system may further play a role, based on its biological features and molecular toolkit.

## Introduction

Since the beginning of the 1990s, there has been an extreme focus in plant molecular biology on one particular model system, the small weed *Arabidopsis thaliana*. *Arabidopsis* offers a combination of characteristics that made it in many ways the perfect model to study plant biology. Besides its obvious advantages as a laboratory model, one of the others was certainly the fact that *Arabidopsis* has a very small genome compared to many other plant species. This was a very important issue in the pre-Next Generation Sequencing (NGS) era, since the sequencing of even a small genome like that of *Arabidopsis* represented at that time a multi-million dollar investment and several years of large scale collaborative efforts (Arabidopsis Genome Initiative, 2000). Moreover, its easy transformation method (Clough and Bent, 1998), and the generation of a large scale functional genomics platform (Alonso et al., 2003) further accelerated the steep rise of *Arabidopsis* to become the gold standard in plant biology.

For many reasons (Scutt and Vandenbussche, 2014) it is obvious that most if not all research subjects studied in plant molecular biology would benefit from the availability of a broader range of experimental systems. With the advent of NGS technology, the sequencing of entire plant genomes and transcriptomes has become technically and financially feasible even for individual research teams, removing one of the major obstacles in the development of new model systems. Consequently, nowadays a wealth of genome sequence data is becoming available, sampled from species throughout plant phylogeny. While a lot can be learned based on the analysis of genomes and transcriptomes alone, the ultimate understanding of the molecular basis of a biological process and its evolutionary origin still relies on classic determination of gene function by loss- and/or gain-of-function approaches. Therefore, the development of gene functional analysis tools for an array of species will be crucial to fully exploit this novel goldmine of sequence information. In recent years, considerable progress has been made with the development of Virus Induced Gene Silencing (VIGS; Burch-Smith et al., 2004; Senthil-Kumar and Mysore, 2011), and TILLING (Wang et al., 2012), allowing comparison of individual gene functions across a broad species pallet of the plant kingdom. Moreover, the recently developed CRISPR/Cas9 technology is creating a revolution in gene functional analysis (Lozano-Juste and Cutler, 2014). The results of such comparative functional studies will form the basis for the understanding of the molecular origin of the diversity of life, one of the most fundamental questions in plant biology and life sciences in general. However, individual genes do not act in an isolated fashion, but are embedded in gene networks. Therefore, studying evolution of gene function ultimately should be put in the context of studying the evolution of the entire gene network, as changes in the function of one gene will in many cases go together with changes in its network environment. Inspired by the famous essay of T. Dobzhansky (Nothing in biology makes sense except in the light of evolution), along the same line one could state that “Comparative gene functional analysis does not make sense except in the light of gene network evolution”. For this reason, novel comparative bio-informatics and systems biology approaches should become progressively more embedded in resolving evolutionary questions. In addition, plant comparative biology/evo–devo will require the availability of a defined set of ‘super’ models occupying key taxonomic positions, in which performing gene functional analysis and testing genetic interactions is as straightforward as, e.g., in *Arabidopsis*, allowing ease of study and comparison of the function of all members of entire gene networks and their genetic interactions.

Here we review why petunia has the potential to become one of these future supermodels. We will first detail its intrinsic qualities as a laboratory model system. Next, we highlight why it is only now that the benefits of the petunia system will become fully exploitable, despite that petunia has already a long history as a model in plant molecular biology and genetics (Gerats and Vandenbussche, 2005). We conclude with a series of arguments in favor of a more diversified multi-model approach in plant biology, and we point out where the petunia model system may further play a role, based on its biological features and (future) molecular genetics toolkit.

## Petunia: Lab-Model Characteristics

**FIGURE 1:**
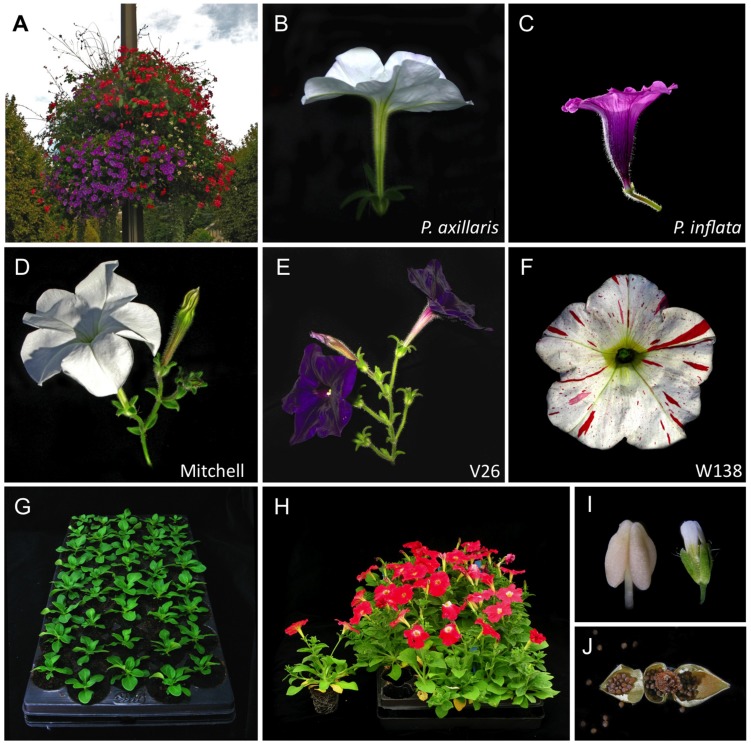
**Petunia species, varieties, and growth characteristics.** **(A)** Petunia is world-wide one of the most popular bedding plants, nowadays frequently used also in hanging baskets for city decoration (Vienne, France). **(B,C)** *Petunia axillaris* and *P. inflata* are two wild species native to South America with contrasting flower architecture, pigmentation, and pollination syndromes (Stuurman et al., 2004). From the early 19th century onward, these two species [or closely related (sub)species thereof] were used as parents in interspecific crosses, providing the genetic material for modern *P. hybrida* cultivars (Stehmann et al., 2009; Segatto et al., 2014). **(D–F)** Most popular petunia varieties used in research. Mitchell and V26 are easily transformable lines; W138 is the high-copy number *dTPH1* transposon line, used in transposon mutagenesis. **(G,H)** The compact growth habit of the W138 variety allows high-density cultivation in tray systems until flowering. **(G)** Young plants; **(H)** Flowering plants; one individual has been sorted from the tray, showing the turfcontainers facilitating plant transfer. **(I)** A single petunia anther fotographed next to an *Arabidopsis* flower illustrates the large size of petunia floral organs. **(J)** Petunia produces dry fruits, each containing ∼60–200 seeds (opened seedpod). Photo credits: **(B)** Peter von Ballmoos; **(C)** Katrin Hermann.

The cultivated garden petunia, with its big colorful flowers and diverse morphology is worldwide one of the most popular bedding flowers (**Figure 1A**). The genus *Petunia*, established as a genus by Jussieu in 1803, originates from South America, and belongs to the family of the Solanaceae (Stehmann et al., 2009). Commercial petunia cultivars as well as the standard laboratory lines have a hybrid origin (therefore called *Petunia hybrida*). Although there has been some discussion on the exact origin of *P. hybrida*, it is generally accepted that crosses in the early 19th century between the white hawkmoth-pollinated *P. axillaris* species (**Figure 1B**) and a member(s) of the purple bee-pollinated *P. integrifolia* group (containing a small number of closely related species, including *P. inflata*; **Figure 1C**) created the basis of the selection material from which all modern *P. hybrida* cultivars are derived (Stehmann et al., 2009; Segatto et al., 2014). Note that because species barriers in the *Petunia* genus are mainly prezygotic, *Petunia* species can be perfectly crossed with each other and yield normal diploid offspring. Thus, *P. hybrida* varieties have the same 2n chromosome numbers as the parental species, and therefore do not suffer from associated genetic complications found in hybrids that are (allo)tetraploid. The most popular petunia lines used in research are V26 and Mitchell, both renowned for their high transformation capacity, and W138, the high-copy number *dTPH1* transposon line used for transposon mutagenesis (**Figures 1D–F**). Petunia laboratory lines display a number of qualities that make petunia ideally suited as a plant model.

### A Short Lifecycle and Easy Culture Conditions

In optimal circumstances, a lifecycle from seed to seed of only 3–3,5 months can easily be obtained, allowing the growth of up to four generations a year. It has been shown for petunia that the rate in progress to flowering increased linearly with increasing temperatures (Adams et al., 1998). In practice, we found that lower temperatures have a double impact on generation time, since they not only delay flowering, but also strongly delay ripening of the seedpods. To reach such a short generation time, we culture petunia at relatively high temperatures (e.g., 27–30°C daytime; 23–25°C night time; long day conditions:15–16 h light/day). Note that the specific growth conditions mentioned above are usually not applied in petunia horticultural production, where energy cost considerations and the aim to obtain a specific plant architecture demand for different growth parameters.

Space usually is a limiting factor in laboratory growth chambers. In contrast to the Mitchell line, which grows very tall and has an inflorescence characterized by very long internodes, the W138 transposon line exhibits a more compact growth habit. It is therefore particularly well suited to cultivation in growth chambers, and can be easily grown in high-density trays until flowering (**Figures 1G,H**). We plant petunia seedlings in trays^1^ (55 cm × 32 cm) containing 40 plants, using turf containers that allow easy repotting of selected individuals later on. Once flowering, we transfer plants of interest in individual containers (0,5 l) and regularly cut side-branches to favor vertical growth, again to save place. With a regular fertilization, plants can in this way easily be maintained for a long period (1–2 years), and may be cut back regularly, while they continue to flower. Petunia grows well in growth chambers, standard green houses, and during spring and summer also outside in simple plastic greenhouse tunnels without any artificial light or temperature control. The latter provides an extremely cheap and feasible solution in case large populations need to be grown, such as for forward genetics screens, and for populations for reverse genetics purposes.

### Easy Propagation, Both Sexual and Asexual

In the wild, petunia depends on animals (bees, hawkmoths, humming birds, depending on the species) for pollination (Stuurman et al., 2004). As a consequence, petunia plants normally do not set seed spontaneously in growth chambers or greenhouses devoid of insects. However, the large petunia flowers and floral organs (**Figure 1I**) make manual pollination (either selfing or crossing) extremely easy. Pollinating a flower requires only a few seconds, each time resulting in a capsule, from which 3–4 weeks later ∼60–200 seeds can be harvested (**Figure 1J**). Asexual propagation by cuttings (massively used in the petunia horticulture) is also very straigthforward. In research, this is particularly useful since it allows the creation of stocks of identical genetic material that can be challenged simultaneously under different conditions. It also allows individual plants of interest to be maintained indefinitely without the need of resowing. Petunia can also be easily grafted (Napoli, 1996), creating a powerful tool to study long distance signaling. Furthermore, propagation by callus culture or plant regeneration starting from leaf explants or protoplasts is also possible.

### Stable/Transient Transformation and Biochemical Analysis

Petunia was among the first plant species that were successfully used to create stable transgenic plants (Horsch et al., 1985). Petunia is classically transformed using a leaf-disk transformation protocol, and a defined set of varieties exists that are particularly easy to transform, such as Mitchell and V26, and the wild species *P. axillaris*. Moreover, any F1 hybrid derived from crossing different petunia varieties displays superior transformation capacity. The latter is routinely applied to transform mutants that arose in W138 (see further), since the pure W138 line is very recalcitrant to transformation.

*Agrobacterium* infiltration in tobacco leaves is widely used in transient assays (Yang et al., 2000). This technique works fine in petunia leaves as well. In addition, due to its large flowers, petals can also be used for infiltration assays (Verweij et al., 2008). Furthermore, an efficient protocol has been developed for the isolation and transformation of protoplasts derived from petals (Faraco et al., 2011). As in tobacco, VIGS works very efficiently in petunia (Chen et al., 2004; Broderick and Jones, 2014). In plant species that are not possible to tranform, VIGS technology often offers the only possible way for gene functional analysis. While VIGS in petunia might offer a rapid way to identify interesting phenotypes, the existing alternatives for gene functional analysis (stable transformation; transposon insertion mutagenesis) might be preferred as a final proof of function. Finally, because of its large leaves and flowers, petunia is particularly suited for biochemical analysis, which often requires large quantities of plant material.

### The Petunia *dTPH1* Transposable Element System in the W138 Line: A Powerful Tool for Forward and Reverse Genetics Approaches

**FIGURE 2:**
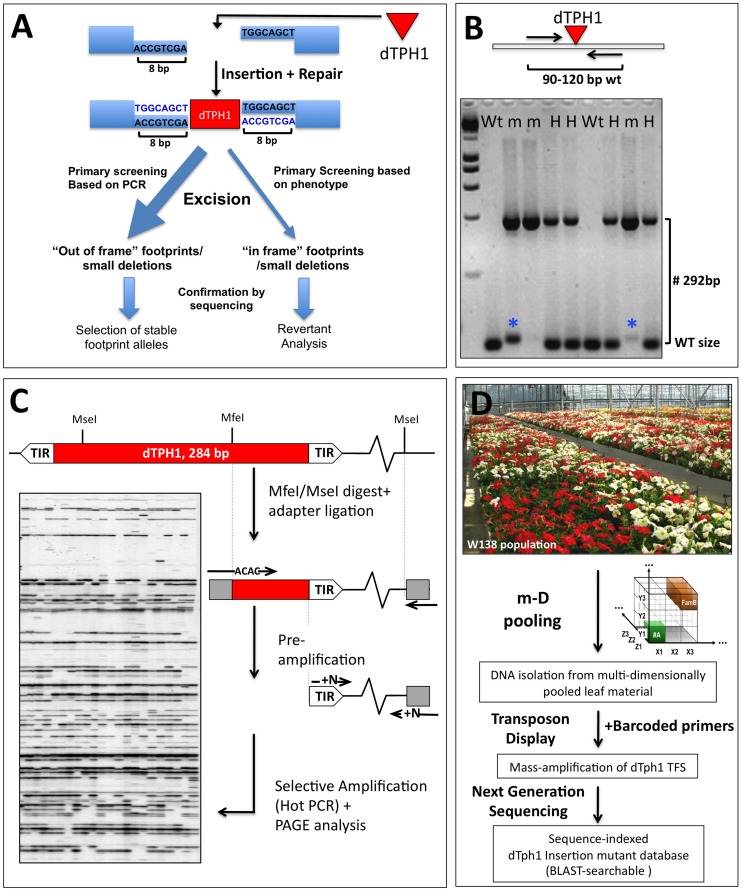
**Petunia *dTPH1* transposon biology and mutagenesis.** **(A)** Petunia *dTPH1* creates a 8 bp target site duplication upon insertion. Excision of the *dTPH1* transposon from an insertion site and subsequent repair may have a variable outcome, resulting in different classes of excision alleles (van Houwelingen et al., 1999). For insertions in the coding sequence, excision will result in an out of frame mutation in most cases, while more rarely, this may result in restoration of gene function (revertant), in case the remaining footprint/deletion is a multiple of three. Note that revertant analysis can be used as a strategy to provide independent proof of gene function. **(B)** The small size of the *dTPH1* transposon allows easy genotyping in a single PCR reaction. A 4% agarose gel is shown loaded with PCR products resulting from amplification with a gene-specific primer pair flanking the insertion site. Fragments containing the *dTPH1* element are 292 bp larger than the WT fragment (284 bp of the transposon + 8 bp target site duplication). Genotypes are indicated Wt = homozygous wild-type; H = heterozygous; m = homozygous mutant. The blue asterisks indicate partial excision of the transposon, resulting in a fragment slightly larger than WT. **(C)** *dTPH1* Transposon Display procedure, used in the forward cloning of *dTPH1* tagged mutants in petunia forward genetics. See Van den Broeck et al. (1998) and Vandenbussche et al. (2013) for more details. **(D)** Procedure for the massive parallel amplification and sequencing of *dTPH1* flanking sequences, derived from populations of 1000–4000 individuals. The resulting sequence database can be BLAST-searched for reverse genetics purposes. For experimental details, see Vandenbussche et al. (2008).

Insertion mutagenesis remains one of the methods of choice to obtain mutants in genes of interest or in hitherto unknown genes. The cloning of the petunia *dTPH1* transposable element (Gerats et al., 1990, 2013) opened the way for insertion mutagenesis approaches in petunia. Interestingly, since this is a completely natural mutagenesis system, the obtained mutants are non-transgenic and therefore their use is not constrained by GMO rules. It turned out that the biology of the *dTPH1* system in the petunia W138 line is extremely well suited for forward and reverse genetics approaches (Koes et al., 1995; Van den Broeck et al., 1998; Vandenbussche et al., 2003, 2008, 2013). The petunia *dTPH1* element is a non-autonomous *hAT*-like transposon that induces a target site duplication of 8 bp upon integretation (**Figure 2A**). Thanks to the small size of the *dTPH1* element (284 bp), genotyping *dTPH1* insertions for segregation analyses is extremely straightforward, and is done in one single PCR reaction using a gene-specific primer pair flanking the insertion site, followed by agarose gel electrophoresis (**Figure 2B**). Note that in practice (partial) excision of the *dTPH1* transposon potentially may complicate the interpretation of PCR genotyping results. Especially homozygous mutants in which partial excision occurs may be wrongly considered as heterozygous plants, since the excision allele is close to WT size. However, this is less of a problem when using 4% agarose gels and choosing segregation primers that generate a WT fragment between ∼90 and 120 bp, resulting in a resolution power that in the majority of the cases clearly distinguishes excision footprints from true WT fragments (**Figure 2B**). Excision of the *dTPH1* transposon from an insertion site and subsequent repair may have a variable outcome, resulting in different classes of excision alleles (van Houwelingen et al., 1999). Depending on the desired effect, this might be exploited in two different ways (**Figure 2A**). Firstly, excision alleles can be selected that cause an out-of-frame mutation in the reading frame, and thus result in a fully stabilized mutant allele. Secondly, although more rarely, excision alleles may be produced that result in the restoration of the reading frame, and possibly gene function. Note that forward phenotypic screening for revertants possibly may require the growing of a large number of plants. Such a revertant analysis associated with the characterization of footprint size can be used to further proof the causal relationship between a *dTPH1* insertion and a phenotype (**Figure 2A**). Note that for insertions in coding sequences in particular, in practice it may often not be needed to stabilize the insertion, since excision events that lead to restoration of gene function are relatively rare. Likewise, the risk of accidentally losing an identified mutation is low.

The *dTPH1* element was isolated from the inbred line W138 (Doodeman et al., 1984), which produces high numbers of new mutations each generation. Interestingly, it was found that the large majority of these new mutations in W138 were caused by *dTPH1* insertions (van Houwelingen et al., 1998; Spelt et al., 2000), despite the presence of other types of transposable elements in petunia. This enormously facilitates the cloning of mutated genes by transposon tagging, since the nature of the mutation (insertion of a *dTPH1* element) can be assumed a priori. For such forward genetics approaches, the *dTPH1* transposon display technique (**Figure 2C**; Van den Broeck et al., 1998; Vandenbussche et al., 2013), or related approaches have been developed, and have led to the identification of many interesting novel genes (e.g., Stuurman et al., 2002; Tobena-Santamaria et al., 2002; Quattrocchio et al., 2006; Cartolano et al., 2007; Rebocho et al., 2008; Verweij et al., 2008; Vandenbussche et al., 2009; Rich et al., 2015).

Furthermore, *dTPH1* inserts preferentially in genic regions and in the W138 line, up to 20–40 novel insertions may arise per individual plant and per generation (Koes et al., 1995; Vandenbussche et al., 2003). These two characteristics together make the W138 line, besides its application in forward genetics, also extremely well suited for reverse genetics mutagenesis: Despite the large (∼1.3 GB) genome size of *Petunia* (Arumuganathan and Earle, 1991), relatively small mutant populations may be sufficient to saturate the genome with genic insertions.

Since in 1990s, W138 populations (varying from 1000 to 4000 plants) have been regularly grown by a handful of petunia groups, providing a source of *dTPH1* mutants for the community, both for forward and reverse genetics screens. For years, reverse genetics screenings of these populations were performed by PCR on a gene-per-gene basis, making the whole procedure slow and labor-intensive (Koes et al., 1995; Vandenbussche et al., 2003). Meanwhile in *Arabidopsis*, a completely different approach was being developed: Insertion sites of a large collection of T-DNA lines were systematically characterized at the sequence level. The resulting publicly available collections of insertion site-sequenced T-DNA lines (Alonso et al., 2003) revolutionized reverse-genetics approaches, since databases with insertion site flanking sequences can be *in silico* searched for mutants of interest based on gene ID or sequence homology, instead of having to perform laborious PCR-based assays. However, because the large scale Sanger sequencing of insertion flanking sequences is very costly, such an approach was the exclusive domain of model organisms financially supported by a large scientific community, such as *Arabidopsis*. From 2005 onward, the first generation of massive parallel sequencing methods started to become available (Margulies et al., 2005), creating a true paradigm shift in molecular biology by bringing large scale sequencing projects financially within reach of individual research teams.

Based on the early GS20 (454) sequencing technology (Margulies et al., 2005), we developed a concept (**Figure 2D**) that allows to mass amplification and sequencing of *dTPH1* transposon flanking sequences (TFS) simultaneously from an entire population, and that permits automatic assignment of TFS to individuals within the same population (Vandenbussche et al., 2008). With this approach, we were able to identify and sequence around 10000 different *dTPH1* insertion loci simultaneously amplified from a population of 1000 individuals. While these results were certainly encouraging and provided for the first time a small blast-searchable mutant collection for petunia, the high costs of GS20 sequencing and limited sequencing capacity were still constraining a large-scale application needed to saturate the genome with *dTPH1* insertions.

## Petuniomics: Petunia Embraces Genomics

### Creation of a Large *dTPH1* Transposon Flanking Sequence Database for Reverse Genetics in Petunia

Since their conception, sequencing capacity of high-throughput sequencing methods has increased exponentially, combined with a steep drop in costs. In particular the Illumina sequencing technology (Bentley et al., 2008) proved to be well adapted to further develop our mass *dTPH1* TFS sequencing principle, leading to a method with unprecedented efficiency, accuracy, and capacity. We are currently preparing a manuscript detailing the protocol and the resulting *dTPH1* TFS collection (Morel et al., unpublished). Analysis of the new *dTPH1* transposon flanking sequence database indicates a good coverage of the petunia genome with *dTPH1* insertions, since we are able to identify (usually multiple) candidate insertions for the large majority of the genes screened for. This collection will revolutionize functional genomics in petunia, in the same way as the SALK collection has revolutionized *Arabidopsis* research. Besides the obvious benefit for research teams using petunia as a model system, this mutant collection can also be of interest for the petunia horticultural industry. Many valuable traits (affecting growth habit, plant architecture, floral architecture) can be obtained by loss-of-function approaches. The non-transgenic mutants identified in our collection can be directly used for crossing with commercial petunia varieties.

### The *Petunia* Genome Sequence(s)

For years, petunia molecular biology research has been slowed down by the unavailability of a sequenced genome. The small size of the petunia scientific community combined with the large size of the *Petunia* genome (∼1.3 GB) rendered a genome sequencing project based on classical Sanger sequencing completely out of reach. As for our insertion mutagenesis program, the advent of NGS technologies suddenly made a petunia genome project feasible. A few years ago, members of the Petunia Platform (see further) joined forces to launch a petunia genome sequencing initiative, in collaboration with BGI (Beijing Genomics Institute, China). To cover the complete gene content of all petunia cultivars, the petunia genome sequencing initiative chose to sequence the genomes of the parental species *P. axillaris* and *P. inflata* (see Petunia: Lab-Model Characteristics), rather than sequencing a few of the many existing hybrids. Sequencing of both *Petunia* species is now finished, and a manuscript is currently being finalized (The Petunia Genome Consortium,in preparation). Consequently, public release of the *Petunia* genome sequences may be expected in the near future. Finally, a number of petunia teams are currently performing a detailed RNAseq-based characterization of the petunia transcriptome in a variety of tissues and processes, which will greatly enhance annotation quality of the genome. Some examples were recently published (Broderick et al., 2014; Villarino et al., 2014; Guo et al., 2015), but many more studies are to be expected.

## Importance of Developing and Maintaining a Broad Range of Plant Models, and the Potential Role of Petunia

While the impact of *Arabidopsis* research on plant biology cannot be overestimated, plant biology will strongly benefit from the development and maintenance of a broader range of plant models. This applies even for research subjects that have been already heavily studied in *Arabidopsis*. Below, we mention five arguments in favor of a more diversified multi-model approach in plant biology. While the first two arguments are so obvious that they do not need further explanation, the last three arguments might be less trivial. Because of the focus of this paper, we provide further support for the last three arguments with specific examples coming from petunia research, but obviously many other examples may be found based on research in other models.

(1) A major challenge in (plant) biology remains to understand the molecular basis of evolution and resulting diversity of lifeforms. It is more than obvious that this question only can be efficiently tackled by comparing gene functions in a diverse set of model species occupying key taxonomic positions and/or exhibiting key evolutionary novelties.
(2) There is an extensive list of biological processes that are too divergent between *Arabidopsis* and other species or that simply do not occur in *Arabidopsis*, and thus require other plant models to analyze. Good examples are fleshy fruit development (tomato), flower color (petunia), myccorrhization, and nodulation (Medicago, Lotus, petunia), leaf and flower development in grasses (rice, maize, *Brachypodium*), floral asymmetry (Antirrhinum), and tuber development (potato) just to name a few.
(3) Plant genomes have a complex origin, shaped by whole and partial genome duplication events that occurred during evolution of the plant lineage, and that were followed by further local rearrangements and duplications. This has been first demonstrated at the whole genome scale in *Arabidopsis* (Arabidopsis Genome Initiative, 2000), and has been confirmed in later sequenced plant genomes. These duplication events have often led to (partial) genetic redundancy. Consequently, many gene functions cannot simply be uncovered by classical forward genetics screens. This is probably the main reason why even now in *Arabidopsis*, despite having been intensively studied for more than two decades, the majority of the genes still remain to be functionally characterized. Note that genetic redundancy in many cases can be efficiently tackled through reverse genetics strategies, but it requires much more effort, and without the guarantee that the result will answer the specific biological question a researcher is interested in. Fortunately, gene duplications and all of the processes that have given a plant genome its current gene content are completely random in nature, meaning that distantly related species will most likely display very different sets of redundant and unique gene functions. Therefore, repeating identical forward genetics screens in distantly related species has the strong potential to uncover completely novel functions that may have remained hidden in redundancy in other species. Good illustrations for this from petunia research are the cases of the *HAM* (*HAIRY MERISTEM*) gene, a promotor of shoot indeterminacy (Stuurman et al., 2002), the auxin biosynthetic gene *FLOOZY* (Tobena-Santamaria et al., 2002), and *MAW* (*MAEWEST)*, a *WOX* gene involved in blade development (Vandenbussche et al., 2009). These genes were all identified based on single mutant phenotypes in forward genetics screens, while similar phenotypes in *Arabidopsis* were only obtained after creating double or higher order mutants (Cheng et al., 2006; Vandenbussche et al., 2009; Engstrom et al., 2011).
(4) Similar to duplications, gene losses may follow independent patterns in different plant lineages. Consequently, some model species may have lost a number of genes that play important roles in other species. An interesting case is at the heart of the ABC model of floral development, of which the corresponding B-class mutants *ap3 (apetala3)* in *Arabidopsis* (Jack et al., 1992) and *def* (*deficiens*) in snapdragon (Sommer et al., 1990) display homeotic conversions of petals into sepals and stamens into carpels. Remarkably, null mutants for the petunia ortholog *PhDEF* (also known as *GREEN PETALS*, *GP*) display only a homeotic conversion of petals into sepals, while stamen development remains unaffected (van der Krol et al., 1993; Vandenbussche et al., 2004). The molecular basis of this one whorled phenotype turned out to be caused not by a difference in the function of *PhDEF* itself, but by the presence of the ancestral B-class gene *TM6* in petunia genome (Rijpkema et al., 2006). *TM6* genes are found in many plant species, but have been lost both in *Arabidopsis* and snapdragon.
(5) Even though the end result of a biological process might be very similar in different species, the underlying regulatory gene networks may have diverged more than generally thought. Some very clear examples of network divergence between *Arabidopsis* and petunia can be found in the mechanisms that pattern homeotic gene expression in the flower (Cartolano et al., 2007), and in the regulation of floral meristem identity genes (Kusters et al., 2015). Thus, even if clear orthologs for all individual components of a regulatory gene network identified in one species can be found back in another species, it does not guarantee that a similar network architecture will control the same biological process in the two species. A better knowledge of the evolution (and divergence) of gene networks may strongly improve the success rate of translating basic knowledge from model organisms to applications in crop species. An improved understanding of gene network evolution could come from the functional comparison of all members of entire gene networks and their genetic interactions in a diverse set of species. Such an approach requires the availability of several ‘super’ models, in which performing gene functional analysis and testing genetic interactions ideally would be as straightforward as in *Arabidopsis*.

While petunia was already renowned as a very convenient plant model, its upcoming large sequence-indexed mutant collection and genome sequence will enormously facilitate gene function analysis at a large scale. Ongoing experiments in our lab involving the comparative functional analysis of ∼30 key floral regulators selected from the *Arabidopsis* gene network indicate that comparative analyses of large regulatory gene networks are indeed feasible in petunia: We succeeded to identify null mutations for >90% of the genes, usually obtaining multiple insertion alleles per gene. Moreover, the straightforward genotyping, crossing and short generation time allow to easily test genetic interactions between mutants: we now regularly obtain double, triple, quadruple and even quintuple mutants.

*Arabidopsis* and petunia belong to the Rosids and Asterids, respectively, which represent the two major groups within the eudicot species, and are thought to have diverged approximately 100 million years ago (Moore et al., 2010). Together with the input from other models, the characterization of gene regulatory networks in petunia and the comparison with *Arabidopsis* will therefore help to reveal the degree of gene network divergence within the higher eudicots.

Petunia belongs to the Solanaceae, which harbors several species that are major (food) crops (potato, tomato, pepper, eggplant, tobacco), while others are mainly cultivated as ornamentals (e.g., petunia, Calibrachoa, Datura, Schizanthus, and many others; Saerkinen et al., 2013). Moreover, some of these food crops have been developed into highly performing plant models, such as tomato (Tomato Genome Consortium, 2012) and potato (Xu et al., 2011). Petunia may be an excellent comparative genetic model to understand the molecular basis and origin of some aspects of the developmental diversity in this family of major agronomical importance. For example, the advanced molecular genetics toolkits available both in petunia and tomato could help to elucidate the molecular mechanisms that determine the difference between dry (petunia) and fleshy (tomato) fruit development (Pabon-Mora and Litt, 2011).

## A Broad Range of Interesting Comparative and Unique Research Topics in Petunia

Nowadays, with its advanced molecular genetics toolkit, petunia is a very attractive model to study a number of subjects (reviewed in the book “Petunia: Evolutionary, Developmental and Physiological Genetics”; Gerats and Strommer, 2009), some of which are difficult to analyze in other species. Reasons for this may be either technical (e.g., other possible species not amenable to reverse and forward genetics) or biological (e.g., a developmental process not occurring in other species).

**FIGURE 3:**
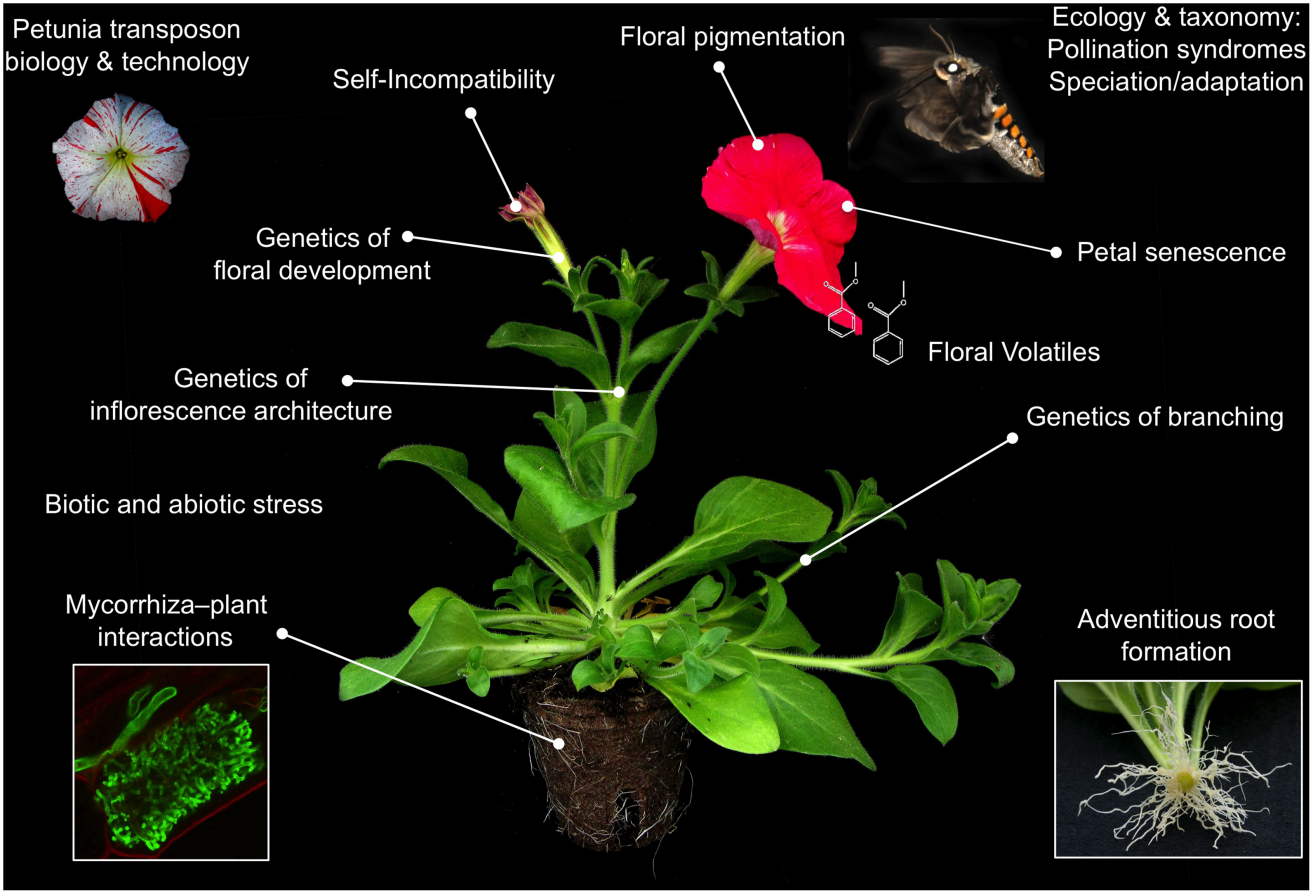
**Overview of popular petunia research topics**.

Petunia development differs in many interesting ways with *Arabidopsis*, which forms an excellent basis for comparative studies, and evo–devo oriented research. The most eye-catching differences are (1) its cymose inflorescence architecture (Kusters et al., 2015), compared to a raceme in *Arabidopsis*; (2) its large, fused and brightly colored petals ideally suited to study flower color (Faraco et al., 2014) and sympetaly (Vandenbussche et al., 2009); and (3) its central placentation topology (Colombo et al., 2008) compared to parietal placentation in *Arabidopsis*. Other interesting differences include the presence of a gametophytic self-incompatibility system (Williams et al., 2015), the existence of different pollination syndromes (Hermann and Kuhlemeier, 2011), its abundant and clock regulated floral volatile production (Verdonk et al., 2003, 2005; Fenske et al., 2015), and being a suited host for myccorrhiza colonization (Rich et al., 2015). In addition, petunia exhibits a more diverse range in forms and ecological niches (Stehmann et al., 2009). Interestingly, since species barriers in petunia are mainly prezygotic, genetic analysis above the ecotype level is much more straigtforward compared to *Arabidopsis* (Nasrallah et al., 2000), and thus allows the integration most of the available molecular tools into ecological studies. Today, research in petunia covers a broad range of topics, many of which have an impact beyond the model system. While a detailed review of the current petunia scientific literature goes beyond the scope of this manuscript, we provide a non-exhaustive list of the most popular subjects in **Figure 3**, which may form a basis for further literature searches. An important part of the research groups working with petunia world-wide are associated with the Petunia Platform^2^, a community driven platform that aims to promote petunia research and to facilitate collaborations among its members. More information on the research performed in petunia can be found on the website, where keyword descriptions of the research of each group are presented, together with links to their respective websites. An ideal introduction into the petunia scientific community is participating in the “World Petunia days” (WPDs), a scientific congress organized every 18 months. Seminar topics traditionally cover molecular mechanisms controlling inflorescence architecture and flower development (floral architecture and floral organ identity), petal senescence, floral scent production, gametophytic self-incompatibility, flower pigmentation, evolution of pollination syndromes, root development (adventitious root formation, mycorrhiza interactions), and petunia genomics, but also new subjects are warmely welcomed. Traditionally, the WPDs have a friendly and informal character, stimulating scientists to exchange ideas, materials, techniques, and unpublished data without any inhibition. Organization of the WPDs is done by volunteering members of the Petunia Platform. Place, date, and organizer of the next edition are announced on the Petunia Platform website.

## Conclusion

For several decades already, petunia has been successfully explored as a model system in plant molecular biology by a relatively small but productive scientific community. Petunia displays a number of characteristics that combined have contributed to its survival as a model system, during a period in which many experimental plant systems were abandoned mainly in favor of *Arabidopsis*. These characteristics include a short generation time, an easy growth habit, its endogeneous highly active transposon system with a strong potential for forward and reverse genetics, an easy transformation protocol and an amenity for biochemical analysis because of its large leaves and flowers. Yet, despite all these advantages, the absence of a genome sequence and the lack of a functional genomics platform equivalent to the *Arabidopsis* SALK collection made working with petunia sometimes feel like driving a F1 racecar without engine. Thanks to NGS technology, both the petunia genome sequence and a large functional genomics platform will become available in the near future. This will finally allow exploitation of the petunia system to its full potential. As a representative of the Asterids, it may be a powerful model to compare the function of entire gene regulatory networks with those of, e.g., *Arabidopsis*, a representative of the Rosids. Belonging to the Solanaceae, petunia may serve as a comparative genetic model in the exploration of the molecular origin of some of the developmental diversity in this family of major agronomical importance. In addition, petunia is expected to further excel in specific research topics that are hard to address in other models.

## Author Contributions

MV wrote the manuscript; PM, PC, and SB contributed to experiments referred to in the manuscript, and commented on the manuscript.

## Conflict of Interest Statement

The authors declare that the research was conducted in the absence of any commercial or financial relationships that could be construed as a potential conflict of interest.

The reviewer Ronald Koes declared a collaboration with the authors Michiel Vandenbussche and Patrice Morel to the handling editor Rainer Melzer, who ensured that the process met the standards of a fair and objective review.

## Footnotes

1 http://www.jiffygroup.com/

2 http://flower.ens-lyon.fr/PetuniaPlatform/PetuniaPlatform.html
